# Cat Eye Syndrome with a Unique Liver and Dermatological Presentation

**DOI:** 10.7759/cureus.37142

**Published:** 2023-04-04

**Authors:** Maureen Mansur, Thomas J Jacob, Helen Wong, Ilya Tarascin

**Affiliations:** 1 Family Medicine, Nassau University Medical Center, New York, USA; 2 Internal Medicine, New York Institute of Technology College of Osteopathic Medicine, Old Westbury, USA

**Keywords:** atopic dermatitis, schmid-fraccaro syndrome, livedo racemosa, primary biliary sclerosis, psoriasis, chatgpt, autoimmune hepatitis, cat eye syndrome

## Abstract

Cat eye syndrome (CES), also known as Schmid-Fraccaro syndrome, is a complex genetic syndrome with a highly variable phenotype that includes ocular coloboma, anal atresia, preauricular skin tags and pits, heart defects, kidney malformations, dysmorphic facial features, and mild to moderate intellectual disability. We describe a case of a 23-year-old male with a past medical history of CES with short stature, mild learning disability, and some dysmorphic facial features who presented with recurrent pruritus and rashes and had mild liver dysfunction. Furthermore, the patient did not have the classic presentation of CES but a clinically milder expression of the phenotypes. Abnormalities in the abdominal ultrasound prompted an ultrasound-guided liver biopsy, which showed bile ductular proliferation with mild portal inflammation composed of lymphocytes and plasma cells, and bridging fibrosis. The patient's labs showed elevated immunoglobulins with the highest increase observed in IgG, along with negative antinuclear antibodies (ANA), negative anti-mitochondrial antibody, and negative hepatitis A/B/C but a weak positive anti-smooth muscle antibody (ASMA). These findings indicated that the patient most likely had autoimmune hepatitis (AIH) or an overlap syndrome with primary sclerosing cholangitis (PSC). The patient was initially treated with steroids and antihistamines for pruritus, which led to some clinical improvement. After dermatological evaluation, the patient was diagnosed with atopic dermatitis and was recently started on a dupilumab 600 mg loading dose and would continue with biweekly dupilumab 300 mg injections. This dermatological finding may require additional examination and can be a unique presentation in patients with CES. This case illustrates that even patients with milder CES expression can experience intense dermatological complications if not effectively managed. CES is a multifactorial disease that requires intervention from multiple specialists. Therefore, primary care physicians must be aware of the potential complications of CES and make adequate referrals to closely monitor patients' symptoms.

## Introduction

Cat eye syndrome (CES), also known as Schmid-Fraccaro syndrome, is a complex disease with highly variable phenotypes; some common presentations include ocular coloboma, anal atresia, preauricular skin tags and pits, heart defects including commonly total anomalous pulmonary venous return, kidney malformations, dysmorphic facial features, and mild to moderate intellectual disability [1]. The vertical coloboma of the iris, after which the "cat eye" syndrome was named, may be absent in 40-50% of cases [2]. Cytogenetic analysis of these patients reveals the presence of supernumerary bi-satellited marker chromosomes, which were derived from an inverted duplication of the short arm (p) and proximal long arm (q) of chromosome 22 (inv dup 22pter-22q11.2) [3]. This results in a tetrasomy (four copies) or trisomy (three copies) of this region on chromosome 22 [4]. Due to the complexity and variability of the disease, it cannot be managed by a single physician. A primary care physician needs to provide the patient with appropriate referrals to different specialists to treat the disease. Early intervention is critical in preventing dangerous sequelae.

## Case presentation

Our patient was a 23-year-old male with a past medical history of CES, chronic transaminitis, pruritus, failure to thrive, and short stature. The patient was delivered at 40 weeks gestation by C-section to a 26-year-old G1P0 mother in the Dominican Republic with a birth weight of 8 pounds. The patient's first word had been spoken only at around 18 months of age, but the remaining developmental history and the ages at which he had achieved milestones were unclear. In 2013, the patient's parents had come to seek care at the family medicine clinic. During the visit, the only complaint initially expressed had been the short stature of the patient; hence, the patient had been referred to a pediatric endocrinologist. Growth hormone deficiency and the short stature homeobox-containing gene (SHOX) DNA sequencing analysis and deletion study was negative. A CGH microarray analysis had shown a 1.1 MB gain at chromosome 22q11.1q11.21 (variant details: arr[hg19]22q11.1q11.21 (16,888,899-17,950,504) x3), indicating a continuous gene duplication syndrome. Sexual precocity-related disorders had been ruled out based on normal testosterone levels and a bone age study.

At his most recent visit, the patient's height (61 inches) and weight (110 lbs) were still not appropriate for his age (23 years). All other vital signs were within normal limits. A review of his systems was positive for abdominal distension and excessive flatulence with a subjective complaint of chronic pruritus. On physical examination, the patient had dry skin, generalized scratch marks over his body, and a soft, non-tender distended abdomen with a 1-cm, palpable hepatomegaly. His rashes would periodically flare up. Around 2018, he experienced an acute flare-up with multiple papules and plaques on the neck, chest, abdomen, back, and bilateral upper and lower extremities. The presentation of the lesions was dry, raised with a pink base, and flaky white that crusted on palpation. Upon observation, his left lower extremity was inflated and bigger than his right lower extremity, for which he was sent to the emergency department and managed with steroids. The diagnosis of this acute episode is unclear as the patient went to a different hospital. In 2023, he was referred to dermatology, where they made a diagnosis of atopic dermatitis, but other differentials are still being considered.

**Figure 1:**
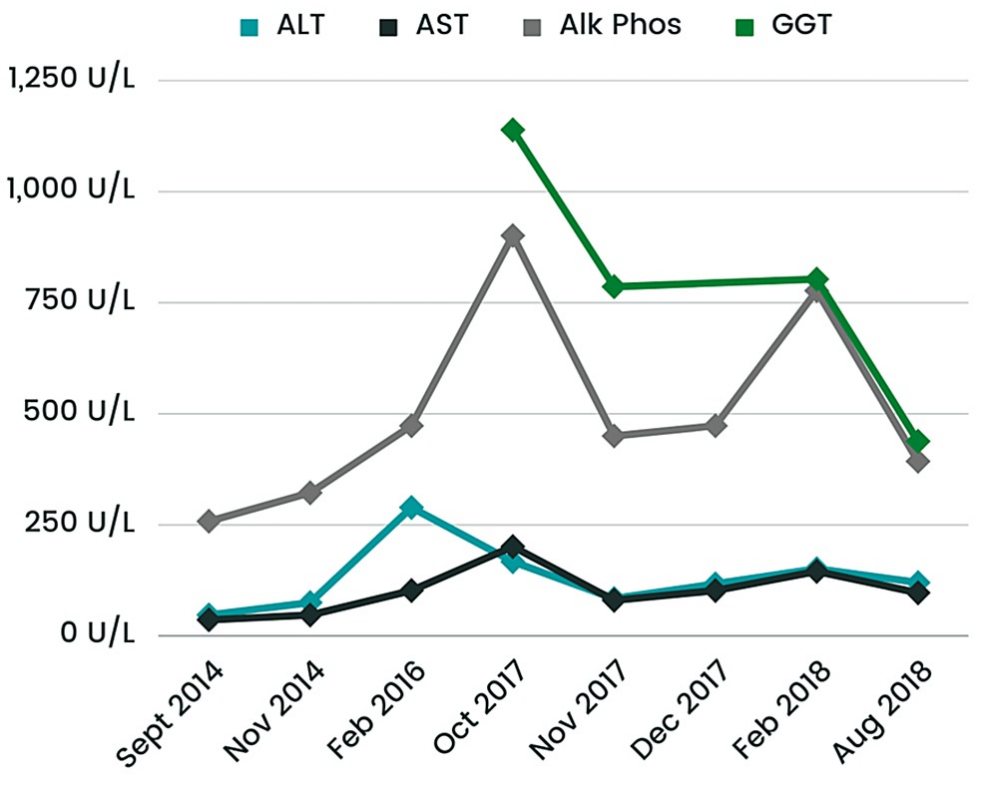
Changes in ALT, AST, ALP, and GGT from 2014 to 2018 ALT: alanine aminotransferase; AST: aspartate aminotransferase; ALP: alkaline phosphatase; GGT: gamma-glutamyl transferase

**Figure 2:**
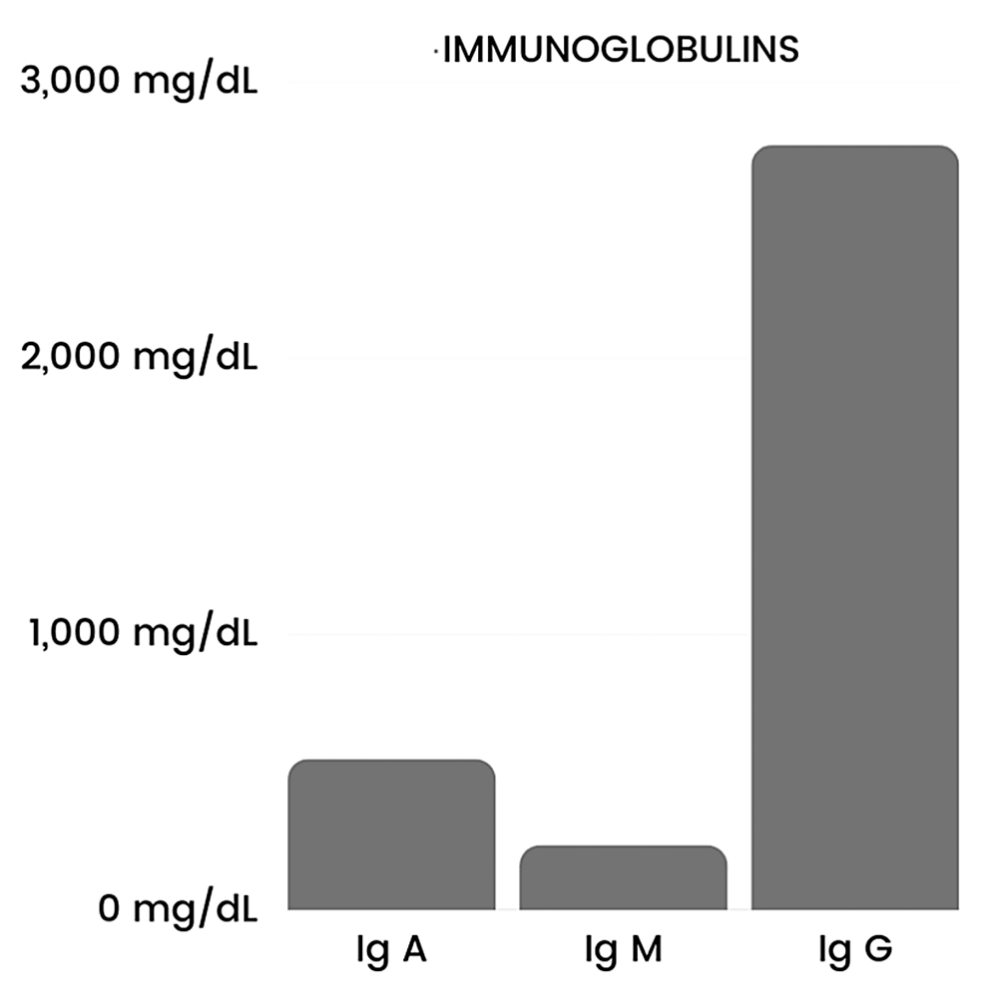
Immunoglobulin A, Immunoglobulin M, and Immunoglobulin G levels

**Figure 3:**
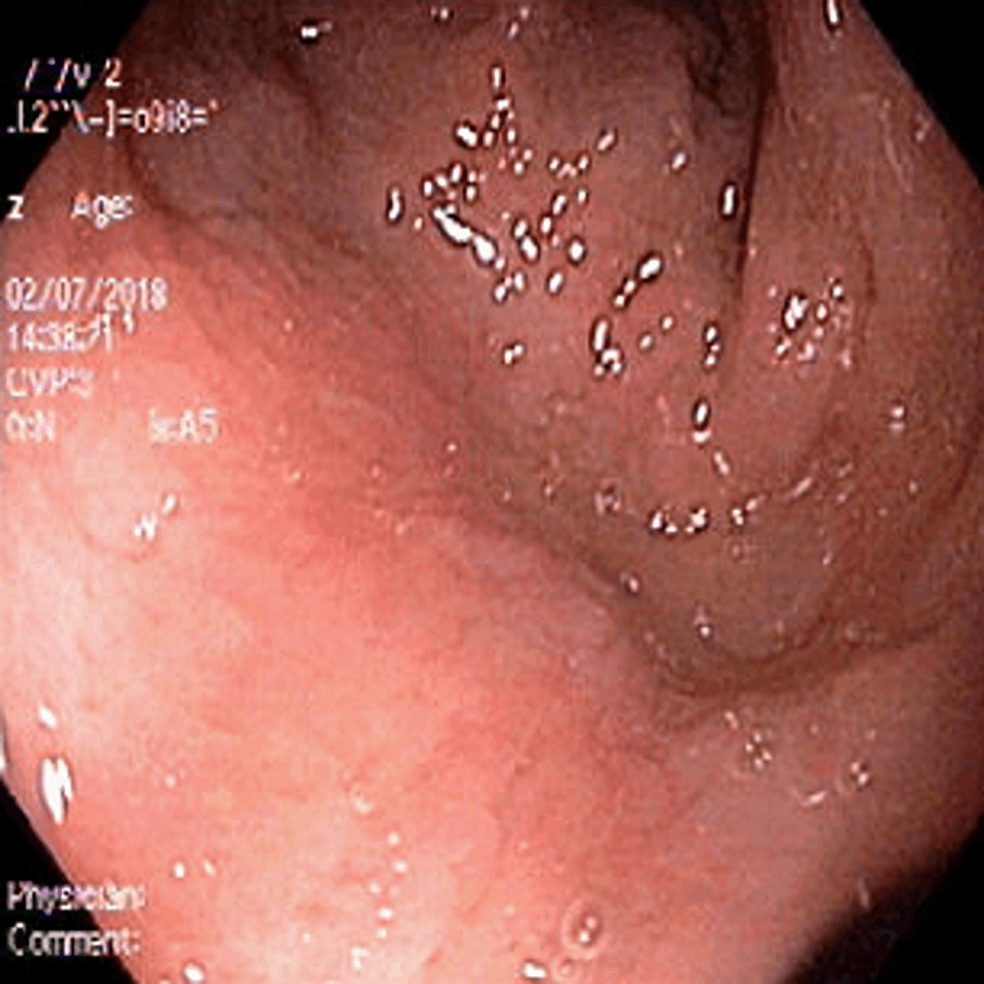
Colonoscopy image indicating erosions at the sigmoid colon

**Figure 4:**
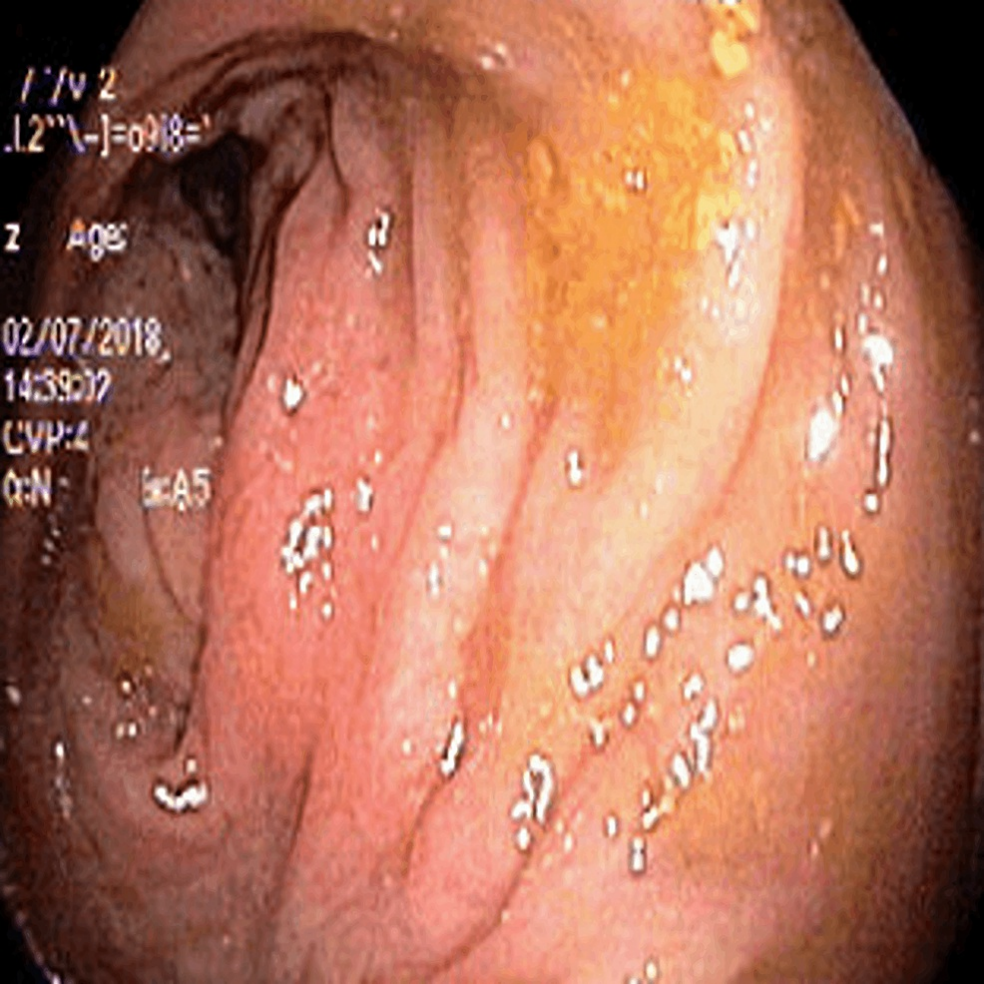
Colonoscopy image indicating an erythematous region of the descending colon

Since CES has many variable presentations, the patient was referred to ophthalmology, cardiology, and a renal ultrasound was requested. The patient was prescribed glasses for bilateral myopia by the ophthalmologist. His cardiac and renal workups were normal including normal EKG and renal and bladder ultrasound scans. However, the patient’s alanine aminotransferase (ALT), aspartate aminotransferase (AST), and alkaline phosphatase (ALP) were significantly elevated around 2014 and were trending higher at each visit (Figure 1). His parents denied any family history of liver disease and his hepatitis A/B/C panels were negative. He was sent for an abdominal ultrasound, which showed increased echogenicity of the portal triads throughout the liver. In 2017, the patient had elevated immunoglobulins with the highest increase in IgG levels (Figure 2), negative antinuclear antibodies (ANA), negative anti-mitochondrial antibody, and a weak positive anti-smooth muscle antibody (ASMA) (titer 1:20). For further evaluation, an ultrasound-guided liver biopsy was performed, which showed bile ductular proliferation with mild portal inflammation and bridging fibrosis. The portal areas exhibited mild inflammation composed of lymphocytes and plasma cells The major differential diagnosis included primary sclerosing cholangitis (PSC), autoimmune hepatitis (AIH), or overlap syndrome, but a diagnosis is still under investigation. Furthermore, an MRI of the liver was done, which showed no intrahepatic biliary ductal dilation and no hepatic parenchymal abnormality. However, the bile duct and common duct morphology were not well evaluated due to artifacts created by patients' excessive movements. He also had an endoscopy and colonoscopy, both showing multiple areas of erythema and erosions (Figures 3, 4). The pathology report of the biopsies taken indicated mild inactive gastritis and mild inactive inflammation of the colon.

The patient was given MiraLAX® for abdominal distention, hydroxyzine 25 mg at bedtime and triamcinolone 0.1% cream for his pruritus, vitamin D 2,000 units daily, and vitamin A 10,000 units daily supplementation. His liver abnormalities were likely due to AIH; however, no treatment was deemed warranted at that point. Since his LFTs, GGT, and ALP were trending down, he was advised to follow up every three months. If there continues to be any significant elevations in his LFTs, steroid treatment regimens would be considered. For further evaluation of liver and abdominal distention, the GI department recommended a new MRCP that needs to be done on an outpatient basis with sedation due to artifacts caused from movement from lack of sedation. Due to the frequent recurrence of the rash, the dermatologist prescribed biweekly dupilumab 300 mg injections after a 600 mg loading dose. The patient was compliant with his medication and injections and reported an improvement in his symptoms.

## Discussion

CES is a genetic disorder that affects various parts of the body and is caused by the duplication of the short arm (p) and proximal long arm (q) of chromosome 22 [5]. CES is named after the characteristic eye appearance seen in some people with this condition, which resembles that of a cat's eye [5]. The symptoms of CES can vary greatly from person to person, and some phenotypes may show coloboma of the iris, anal atresia with fistula, down-slanting palpebral fissures, preauricular pits, and/or tags, mild developmental delays, and renal and heart malformations (anomalous pulmonary venous return) [4,6]. The severity of CES can range from mild to severe, and there is no cure for this condition. Treatments usually focus on managing the symptoms and may include surgery to correct eye abnormalities, as well as speech therapy, language therapy, special education, and other supportive measures [5].

The classic triad of presentations in patients with CES comprises iris coloboma, anal atresia, and preauricular skin tag or pit [7]. Our patient had a variable presentation because he did not have many of the debilitating congenital phenotype expressions except for short stature, some dysmorphic facial features, liver dysfunction, and intellectual disabilities, which led to a delayed diagnosis. However, this case illustrates the potential for developing liver and severe dermatological complications in patients with CES. There is currently no specific association between CES and rashes [5]. However, some individuals with CES may have skin issues or rashes as a result of other underlying health problems [5]. Our initial differential diagnosis of the pruritus, which later developed into a rash, included pruritus secondary to liver dysfunction and psoriasis. The patient's clinical presentation of the rash did not fit our differential diagnosis, even though it was managed with steroids and antihistamines.

Our patient also had chronically elevated ALT, AST, ALP, and IgG levels with a weak positive ASMA. After ruling out viral hepatitis, drug-induced hepatitis, alcohol-induced hepatitis, Wilson’s disease, hereditary hemochromatosis, and alpha-1 antitrypsin deficiency, the two main differentials were AIH and PSC. AIH is a chronic liver disease that occurs when the body's immune system mistakenly attacks liver cells, leading to inflammation and injury to the liver [5]. The main diagnostic criteria for AIH are as follows: one elevated serum aminotransferase at least twice the upper limit of normal; a minimum of one positive laboratory test (increased total IgG, ANA, or ASMA); and the exclusion of other diseases with a similar presentation [7]. The Revised Original Scoring System of the International Autoimmune Hepatitis Group can also be used to aid in the diagnosis of AIH; a pretreatment aggregate score greater than 15 signifies a definitive diagnosis, while a score of 10-15 indicates a probable diagnosis (patient score: 15) [7]. Upon further evaluation, our patient’s ultrasound-guided liver biopsy showed bile ductular proliferation with mild portal inflammation and bridging fibrosis. The portal areas demonstrated mild inflammation composed of lymphocytes and plasma cells. The predominance of periductal fibrosis is a histopathological feature of PSC [8]. Thus, the patient had findings associated with both AIH and PSC. Establishing the diagnosis of AIH-PSC is difficult; however, patients with AIH-PSC generally have an earlier onset (mean age of 21 years), higher aminotransferase and serum immunoglobulin levels, and prevalence of autoantibodies [9]. These findings suggest that our patient possibly had an overlap of PSC and AIH. A combination of corticosteroids and azathioprine has been shown to reduce relapses and withdrawal to treatment compared to using corticosteroids alone [10]. Adding ursodeoxycholic acid (UDCA) to the corticosteroid regimen in AIH-PSC overlap patients has been shown to increase survival rates [8]. Since our patient remains asymptomatic, no treatment is indicated at this time.

There is no mention of a specific dermatological rash limited to CES in the current literature, but further research is needed to explore this clinical feature. Our patient presented with a rash that seemed psoriatic at first glance; however, we believe that the pathophysiology of this specific rash does not match that of psoriasis. A diagnosis of psoriasis is normally made through physical examination, where well-demarcated, erythematous plaques with coarse-scale would be seen. However, due to the extensive surface area coverage of our patient’s rash and since it was controlled with antihistamines and corticosteroids, we do not believe he had psoriasis based on the treatment response. Had it been psoriasis, his extensive rash would designate a diagnosis of at least moderate to severe psoriasis, for which a treatment involving phototherapy or systemic therapy with retinoids, methotrexate, cyclosporine, or biologic agents would be required [11].

The main skin finding associated with the gene segment involving CES is a livedoid rash stemming from a deficiency of adenosine deaminase 2 (DADA2) or Sneddon syndrome [12]. DADA2 results from autosomal recessive mutations of the CECR1 (Cat Eye Syndrome Chromosome Region 1) gene found on chromosome 22q11.1, but it is a separate autoinflammatory disease characterized by immunodeficiencies, systemic inflammation, and early-onset stroke, which were not seen in our patient. The skin manifestations in DADA2 are livedo racemosa, a bluish net-like discoloration with no papules, painful cutaneous nodules, and erythematous papules [12].

Another differential considered was mycosis fungoides, a subtype of cutaneous T-cell lymphoma. It commonly presents with pruritic, scaly patches or plaques and rarely with generalized erythroderma, hypopigmented or hyperpigmented lesions, papules, or generalized erythroderma [13]. A definitive diagnosis is made through a skin biopsy, clinical criteria, immunopathological criteria, and molecular biological criteria, but some patients may never progress to that point due to the resolution of skin findings with topical corticosteroids. We have not biopsied our patient yet, and hence we cannot rule out this possibility. The peak incidence of this disease occurs at ages 55-60 years and is more common in black populations, which do not overlap with our patient’s demographic background [14].

Our patient was diagnosed with moderate atopic dermatitis by a dermatologist and is currently being treated with dupilumab. A case of eczema in CES was described previously in a patient with a more severe phenotypic presentation and an unusually larger trisomy and a smaller tetrasomy of proximal 22q11 [15]. Eczema described in the case reported by Fekete et al. resulted from severe hypogammaglobulinemia leading to long infection periods caused by Staphylococcus aureus with grave palmar and plantar eczema. Unlike this patient presented by Fekete et al., our patient lacks similar abnormal genetic findings and severe phenotypic features like cardiac defects, musculoskeletal abnormalities, severe immunodeficiency, epilepsy, and malformation of the colon [15]. We do not believe that our patient’s rash is a result of skin infections, and in contrast, our patient had elevated immunoglobulins. The discordance in the severity of this rash compared to the other relatively benign physical findings this patient has begs the question as to whether moderate to severe eczema can be included as one of the presentations of CES. Follow-up on treatment outcomes and further studies should be conducted to see if this diagnosis is accurate and not an atypical presentation of other conditions. Also, further research into the association between autoimmunity and this specific segment of chromosome 22 should be conducted.

## Conclusions

CES is a condition that cannot be cured but can be managed with the help of specialists in different fields. This case demonstrates that in addition to various other clinical presentations, patients with CES can also develop liver dysfunction due to an overlap between AIH and PSC, and severe dermatological findings. Close monitoring of liver function and dermatological findings is necessary to prevent the development of complications. Additionally, further research is needed on CES and its dermatological associations.

## Figures and Tables

**Figure 5:**
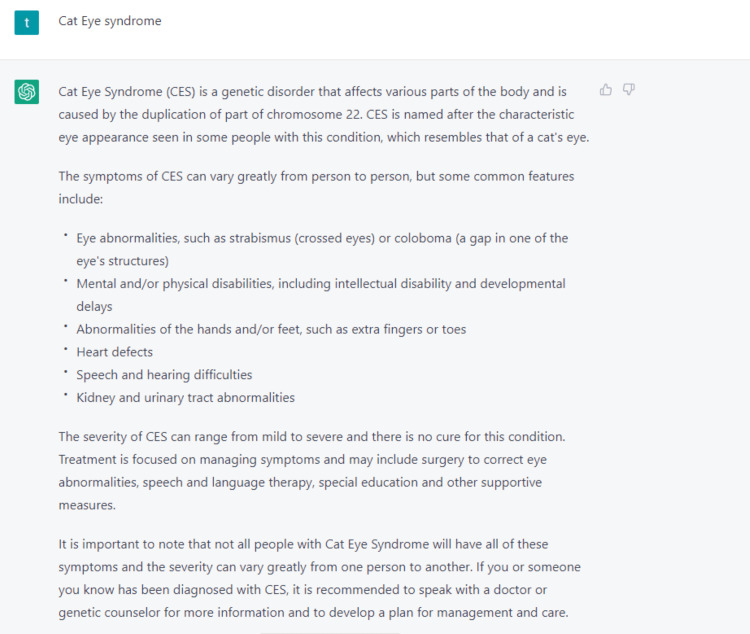
ChatGPT results on features and treatments of cat eye syndrome

**Figure 6:**
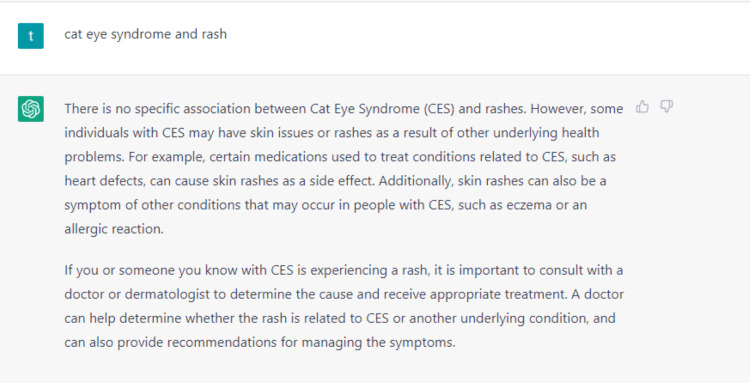
ChatGPT findings on dermatological associations of cat eye syndrome

**Figure 7:**
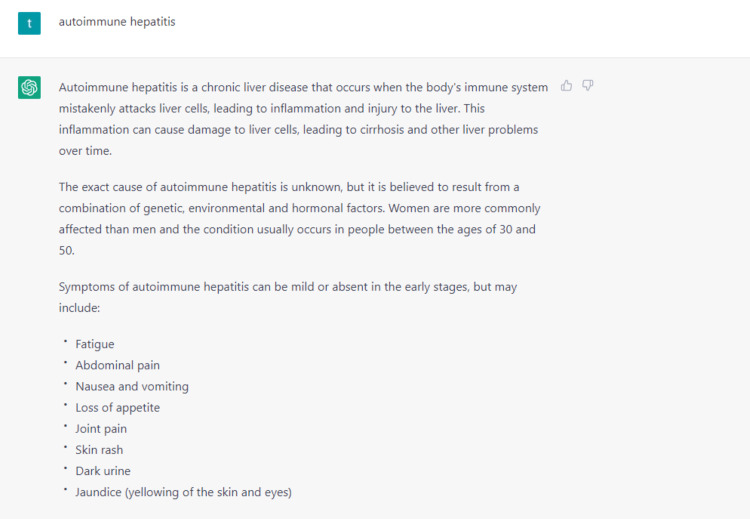
ChatGPT results on the classical presentations of autoimmune hepatitis
